# Supplementary material for: Single-atom catalysts reveal the dinuclear characteristic of active sites in NO selective reduction with NH3
Source: Nat Commun. 2020 Mar 24;11:1532. doi: 10.1038/s41467-020-15261-5 (PMC7093475; doi:10.1038/s41467-020-15261-5)
Supplement: Supplementary file 1 — Supplementary Information [file 41467_2020_15261_MOESM1_ESM.pdf]

Supplementary Information

Single-Atom Catalysts Reveal the Dinuclear Characteristic of Active Sites in NO Selective Reduction with NH_3

Qu et al.

Contents

Supplementary Discussion **3**

The structure of α-Fe_2O_3 nanosheet 3

Calculation on the number of the anchoring sites 3

Analysis of the STEM image 4

H_2-TPR. 4

Supplementary Tables **6**

Supplementary Table 1. EXAFS analysis results of the samples 6

Supplementary Table 2. The calculated area ratio of sub-bands derived from the deconvoluted H_2-TPR profiles of α-Fe_2O_3 and Mo_1/Fe_2O_3 7

Supplementary Figures **8**

Supplementary Figure 1. SXRD patterns of Mo_1/Fe_2O_3, α-Fe_2O_3, and α-MoO_3 8

Supplementary Figure 2. TEM, HRTEM images and structure model of α-Fe_2O_3 nanosheets 9

Supplementary Figure 3. AC-STEM image of Mo_1/Fe_2O_3 10

Supplementary Figure 4. XANES spectra of samples 11

Supplementary Figure 5. R-space and inverse FT spectra of samples 12

Supplementary Figure 6. Raman spectra of samples 13

Supplementary Figure 7. Mo 3*d* XPS of samples 14

Supplementary Figure 8. Fe 2*p* XPS of samples 15

Supplementary Figure 9. DRIFT spectra of NH_3 adsorption on samples at 50°C 16

Supplementary Figure 10. DRIFT spectra of NH_3 adsorption on samples at 250°C 17

Supplementary Figure 11. X_{NO} as a function of temperature over Mo_1/Fe_2O_3 with the different Mo loadings together with α-Fe_2O_3 and α-MoO_3 18

Supplementary Figure 12. SCR performance as a function of temperature over Mo_1/Fe_2O_3 and α-Fe_2O_3 19

Supplementary Figure 13. Effect of H_2O and SO_2 on catalytic activity over Mo_1/Fe_2O_3 20

Supplementary Figure 14. Arrhenius plots of NO conversions over Mo_1/Fe_2O_3 with different Mo loadings 21

Supplementary Figure 15. X_{NO} as a function of temperature over Mo_1/Fe_2O_3 and 3.3% Mo/Fe_2O_3 22

Supplementary Figure 16. SXRD patterns of $Fe_2(MoO_4)_3$, 3.3% Mo/Fe_2O_3, and α-Fe_2O_3 23

Supplementary Figure 17. TOFs in SCR at 270 ºC over Mo_1/Fe_2O_3 with different Mo loadings .. 24

Supplementary Figure 18. XRD patterns of W_1/Fe_2O_3, α-Fe_2O_3, and γ-WO_3 25

Supplementary Figure 19. AC-STEM image of W_1/Fe_2O_3 26
Supplementary Figure 20. H_2-TPR profiles of W_1/Fe_2O_3, Fe_1/WO_3, α-Fe_2O_3 and γ-WO_3.. 27
Supplementary Figure 21. X_{NO} as a function of temperature over W_1/Fe_2O_3 with the different W loadings and α-Fe_2O_3 28
Supplementary Figure 22. Arrhenius plots of NO conversions over W_1/Fe_2O_3 with different W loadings 29
Supplementary Figure 23. X_{NO} as a function of temperature over Mo_1/Fe_2O_3, W_1/Fe_2O_3, and Fe_1/WO_3 30
Supplementary Figure 24. TOFs over Mo_1/Fe_2O_3, W_1/Fe_2O_3, and Fe_1/WO_3 at 270 ºC........ 31
Supplementary Figure 25. TEM images, structure model and EDX mappings of Fe_1/WO_3 32
Supplementary Figure 26. X_{NO} as a function of temperature over Fe_1/WO_3 with the different Fe loadings and γ-WO_3........ 33
Supplementary Figure 27. The deconvoluted H_2-TPR profiles of Mo_1/Fe_2O_3 and α-Fe_2O_3 34
Supplementary References 35

Supplementary Discussion

The structure of α-Fe_2O_3 nanosheet. The SXRD pattern of the α-Fe_2O_3 nanosheet in Supplementary Figure 1 is indexed to rhombohedral Fe_2O_3 (JCPDS 33-0664) with $a = b = 5.036$ Å and $c = 13.749$ Å. According to the TEM/HRTEM images (Supplementary Figure 2), the synthesized α-Fe_2O_3 has a hexagonal nanosheet-shaped morphology with average sizes of 90 nm in width and 9 nm in thickness. In Supplementary Figure 2C, two series of fringes with an intersection angle of 60° are ascribed to (-120) and (110) planes, respectively, from which the basal up surfaces of the horizontally-lying nanosheets are deduced to be α-Fe_2O_3(001) surface. Thus, the proportion of exposed (001) surfaces is calculated to be ~90% as follows: 2 × (90 + 180) × 45√3 / [2 × (90 + 180) × 45 √3 + 6 × 90 × 9] = 90%.

Calculation on the number of the anchoring sites. For α-Fe_2O_3, the number of anchoring sites on (001) surfaces is calculated as follows:

(i) *Average density of the unit cell of the Fe_2O_3.* For the perfect Fe_2O_3 crystal, the unit cell can be expressed as a formula $Fe_{12}O_{18}$, and thus the mass (M_{cell}) of one unit cell is calculated to be 1.6×10^{-21} g. The volume (V_{cell}) of one unit cell according to the lattice parameters ($a = b = 0.503$ nm, $c = 1.374$ nm) and the hexagonal structure feature of the Fe_2O_3 (the SXRD pattern of the Fe_2O_3 in Supplementary Figure 1) can be calculated:

$V_{cell} = \sqrt{3}/2 \times a^2 \times c = \sqrt{3}/2 \times (0.503\ \text{nm})^2 \times 1.374\ \text{nm} = 0.301\ \text{nm}^3$

Thus, an average density (ρ) of the unit cell is calculated:

$\rho = M_{cell} / V_{cell} = 5.316 \times 10^{-21}\ \text{g nm}^{-3}$.

(ii) *The number of the nanosheets for 1 g Fe_2O_3 nanosheets.* The volume (V_{sheet}) and mass (M_{sheet}) of single Fe_2O_3 nanosheet are calculated according to the TEM/HRTEM images and a corresponding model (Supplementary Figure 2) as follows:

$V_{sheet} = 3\sqrt{3}/2 \times l^2 \times h = 3\sqrt{3}/2 \times (90\ \text{nm})^2 \times 9\ \text{nm} = 1.90 \times 10^5\ \text{nm}^3$,

$M_{sheet} = V_{sheet} \times \rho = 1.90 \times 10^5\ \text{nm}^3 \times 5.316 \times 10^{-21}\ \text{g/nm}^3 = 1.01 \times 10^{-15}$ g,

where l and h represent the average length and height of the Fe_2O_3 nanosheets, respectively (Supplementary Figure 2). Therefore, the number (N_{sheet}) of the Fe_2O_3 nanosheets that 1 g catalyst (M_{cat}) contains:

$N_{sheet} = M_{cat} / M_{sheet} = 1\ \text{g} \div (1.01 \times 10^{-15}\ \text{g}) = 9.90 \times 10^{14}$.

(iii) *The number of the anchoring sites on the (001) surfaces of 1 g Fe_2O_3.* According to the model in Supplementary Figure 2D, the areas of the single anchoring site (S_a) and the (001) surface of 1 g Fe_2O_3 (S_{001}) can be respectively calculated as:

$S_a = \sqrt{3}/2 \times a^2 = 0.219\ \text{nm}^2$,

$S_{001} = 2 \times 3\sqrt{3}/2 \times l^2 \times N_{sheet} = 2 \times 3\sqrt{3}/2 \times (90\ \text{nm})^2 \times 9.90 \times 10^{14} = 4.17 \times 10^{19}\ \text{nm}^2$.

Therefore, the number of the anchoring sites (N_a) can be calculated as follows:

$N_a = S_{001} / S_a = 4.17 \times 10^{19}$ nm^2 / 0.219 nm^2 = 1.9×10^{20}.

Based on the above discussion, if all the anchoring sites are occupied by Mo or W ions, the Mo or W loading in weight with respect to Fe_2O_3 is ∼3.0 wt% or ∼5.8 wt%, respectively, according to the following calculations:

Mo wt% = (N_a/N_A) mol × M_{Mo} g mol^{-1} / (1 g) = (1.9×10^{20} / (6.02×10^{23}) mol × 95.94 g mol^{-1} /(1 g) = 3.0 wt%;

W wt% = (N_a/N_A) mol × M_W g mol^{-1} / (1 g) = (1.9×10^{20} / (6.02×10^{23}) mol × 183.8 g mol^{-1} /(1 g) = 5.8 wt%;

The number of anchoring sites and the loading of Fe on the γ-WO_3 nanosheets can also be calculated by the same method according to the structures of γ-WO_3 and the dinuclear sites (Supplementary Figures 17 and 22). If all the anchoring sites are occupied by Fe ions, the Fe loading in weight with respect to γ-WO_3 is ~0.42%.

Analysis of the STEM image. In AC-STEM, the intensity contributed by an atom is approximately proportional to Z^n (where Z is the atomic number, $1.6 < n < 1.9$)[1], and thus the intensity is greater for heavier atoms. The brighter points in Figure 1a were identified as Mo atoms and the intensity ratio of the Mo atom to Fe atom is theoretically in a range 2.1-2.5 [$(Z_{Mo}/Z_{Fe})^n = (42/26)^n$, $1.6 < n < 1.9$]. Then, we calculated the intensity ratio of the brighter dots to the darker ones according to the AC-STEM image in Figure 1 and Supplementary Figure 3. Considering that the number of atoms in a column can also have an effect on the intensity[2], the background intensity was subtracted. Therefore, the experimental intensity ratio is ∼2.3 [(210-50)/(120-50) = ∼2.3], in the theoretical intensity ratio range 2.1-2.5. This result indicates that the brighter points in Figure 1a are Mo atoms and the lighter gray points are Fe atoms.

H_2-TPR. The reduction of α-Fe_2O_3 starts at ~190 °C, and a weak reduction peak at ~267 °C can be ascribed to the surface active oxygen species, merely accounting for 2% of the total oxygen species of α-Fe_2O_3. According to the calculated area ratios of the sub-bands at relatively low (I), medium (II) and high (III) temperatures, which was 7.8 : 22.2 : 70.0 (Supplementary Table 2 and Supplementary Figure 27), i.e., 0.7 :2 : 6, Fe species in α-Fe_2O_3 mainly followed a three-step reduction process: $Fe_2O_3 \rightarrow Fe_3O_4 \rightarrow FeO \rightarrow Fe$. During the whole process, the H_2 consumption ratio of the three steps should be 1 : 2 : 6. Note that the H_2 consumption ratio at the low temperature (I) was smaller than the theoretical value of the first reduction step ($Fe_2O_3 \rightarrow Fe_3O_4$), indicating the existence of the surface defect oxygen. According to the H_2 consumption ratio at the low temperature (I), an actual formula of α-Fe_2O_3 nanosheet should be $Fe_2O_{2.9}$.

For Mo_1/Fe_2O_3, there was also a weak reduction peak at ~267 °C, which accounts for 2% of the total oxygen species of Mo_1/Fe_2O_3. This result indicated that the Mo anchoring has little effect on the redox

ability of the surface active O atoms of Mo_1/Fe_2O_3. However, the reduction temperatures of the bulk O atoms shifted up by ~20 °C. We further calculated H_2 consumption area ratios of I : II : III, which are also 0.7 : 2 : 6, suggesting that the three-step reduction mechanism was not influenced by the Mo loading. Furthermore, an extra weak peak appears in the high temperature regime 460-615 °C, which accounts for 1.8% of the total oxygen species of Mo_1/Fe_2O_3. We calculated the ratio of the consumed hydrogen atoms of this peak to Mo atoms (H/Mo) to be ~5, comfirming the reduction process of $Mo^{5+} \rightarrow Mo^0$.

Supplementary Tables

Supplementary Table 1. EXAFS analysis results of the samples.

Sample	Shell	CN^{a}	R^{b} (Å)	$\sigma^{2\,c}$(Å^2)	$\Delta E_0{}^{d}$
Mo_1/Fe_2O_3 ^{e}	Mo-O	6	1.88(7)	0.008(4)	-5.4
	Mo-Fe	1	2.86(4)	0.005(4)	-3.4
	Mo-Fe	3	2.93(7)	0.039(5)	+0.4
Fe_2O_3 ^{f}	Fe-O	6	1.98(1)	0.010(3)	-5.1
	Fe-Fe	4	3.02(1)	0.010(6)	+3.1
	Fe-Fe	3	3.36(0)	0.001(3)	+4.9

^{a}CN, coordination number;

^{b}R, distance between absorber and backscatter atoms;

$^{c}\sigma^2$, Debye-Waller factor;

$^{d}\Delta E_0$, energy shift;

^{e}R-space fit, Δk = 2.3–11.4 Å^{-1}, Δr = 0.8–3.2 Å;

^{f}R-space fit, Δk = 2.5–9.0 Å^{-1}, Δr = 0.4–4.1 Å;

Supplementary Table 2. The calculated area ratio of sub-bands derived from the deconvoluted H_2-TPR profiles of α-Fe_2O_3 and Mo_1/Fe_2O_3.

Sample	Area ratio I (%)	Area ratio II (%)	Area ratio III (%)	Area ratio IV (%)
Fe_2O_3	$I_{267}+I_{300}$ = 7.8	II_{340} = 22.2	$III_{368}+III_{400}+III_{426}$ = 70.0	-
Mo_1/Fe_2O_3	$I_{267}+I_{321}$ = 7.9	II_{366} = 22.8	$III_{404}+III_{439}$ = 67.5	IV_{532} = 1.8

Supplementary Figures

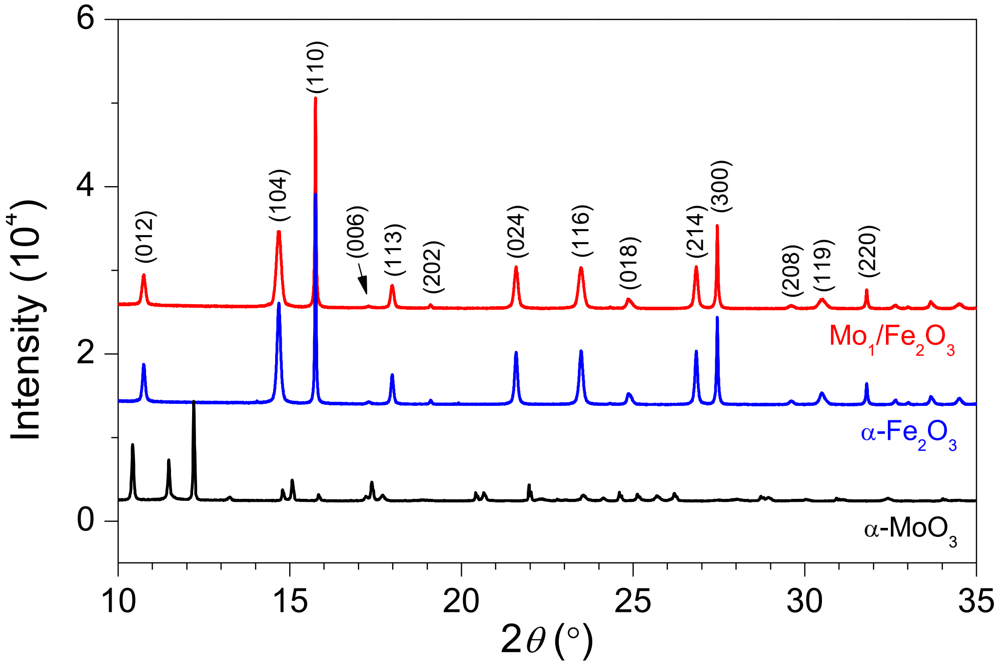

Supplementary Figure 1. SXRD patterns of Mo_1/Fe_2O_3 (red line), α-Fe_2O_3 (blue line), and α-MoO_3 (black line).

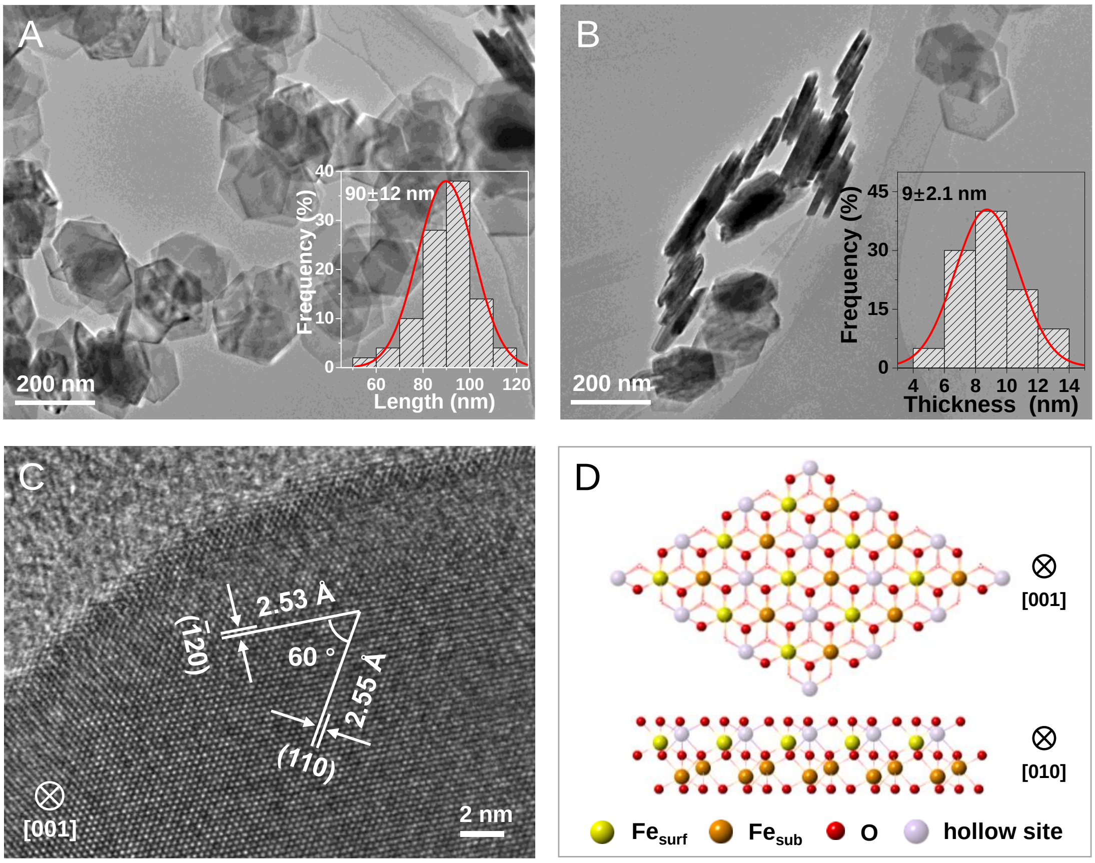

Supplementary Figure 2. (**A,B**) TEM and (**C**) HRTEM images of α-Fe_2O_3 nanosheets. Insets in panels **A** and **B** are the side length and thickness distributions of the α-Fe_2O_3 nanosheets, respectively. (**D**) Structural model of α-Fe_2O_3 nanosheets. The yellow, brown, red, and translucent balls represent surface Fe atoms, subsurface Fe atoms, O atoms, and three-fold hollow sites, respectively.

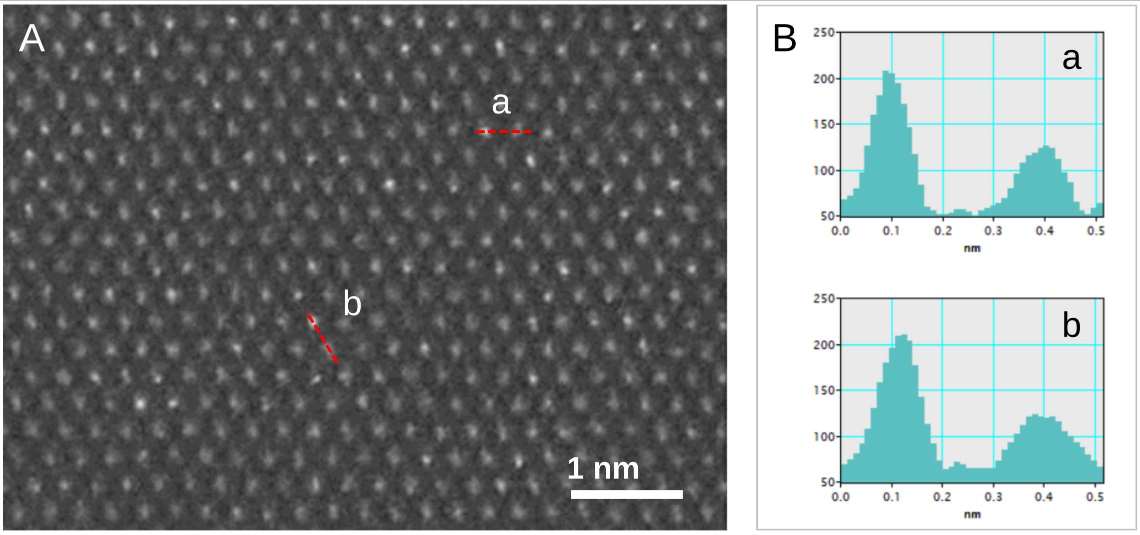

Supplementary Figure 3. (**A**) AC-STEM image of Mo_1/Fe_2O_3. (**B**) The image intensities of the dinuclear sites in directions a and b shown in **A**.

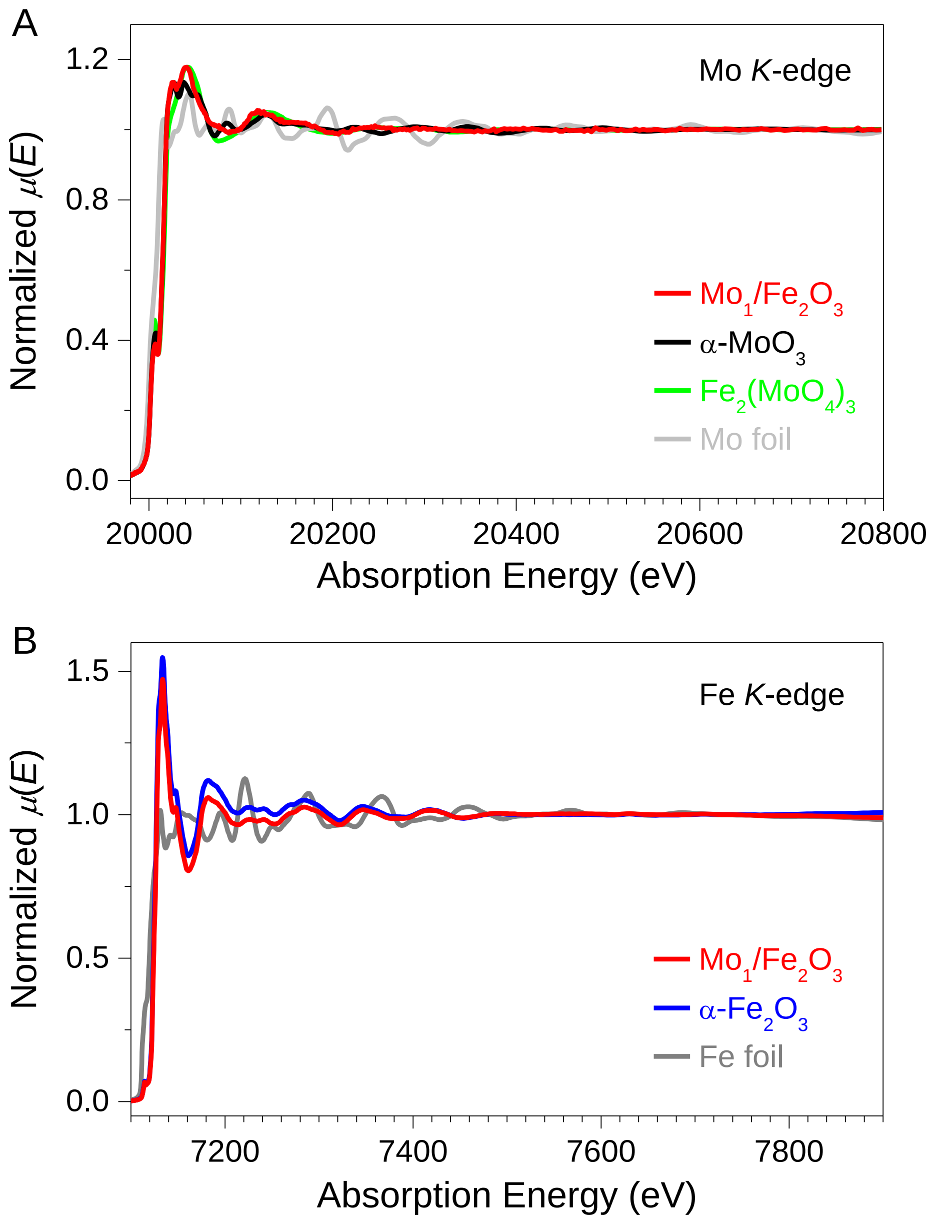

Supplementary Figure 4. (**A**) X-ray absorption spectra of Mo_1/Fe_2O_3 (red line), α-MoO_3 (black line), $Fe_2(MoO_4)_3$ (green line), and Mo foil (gray line) at the Mo *K*-edge. (**B**) X-ray absorption spectra of Mo_1/Fe_2O_3 (red line), α-Fe_2O_3 (blue line), and Fe foil (gray line) at the Fe *K*-edge.

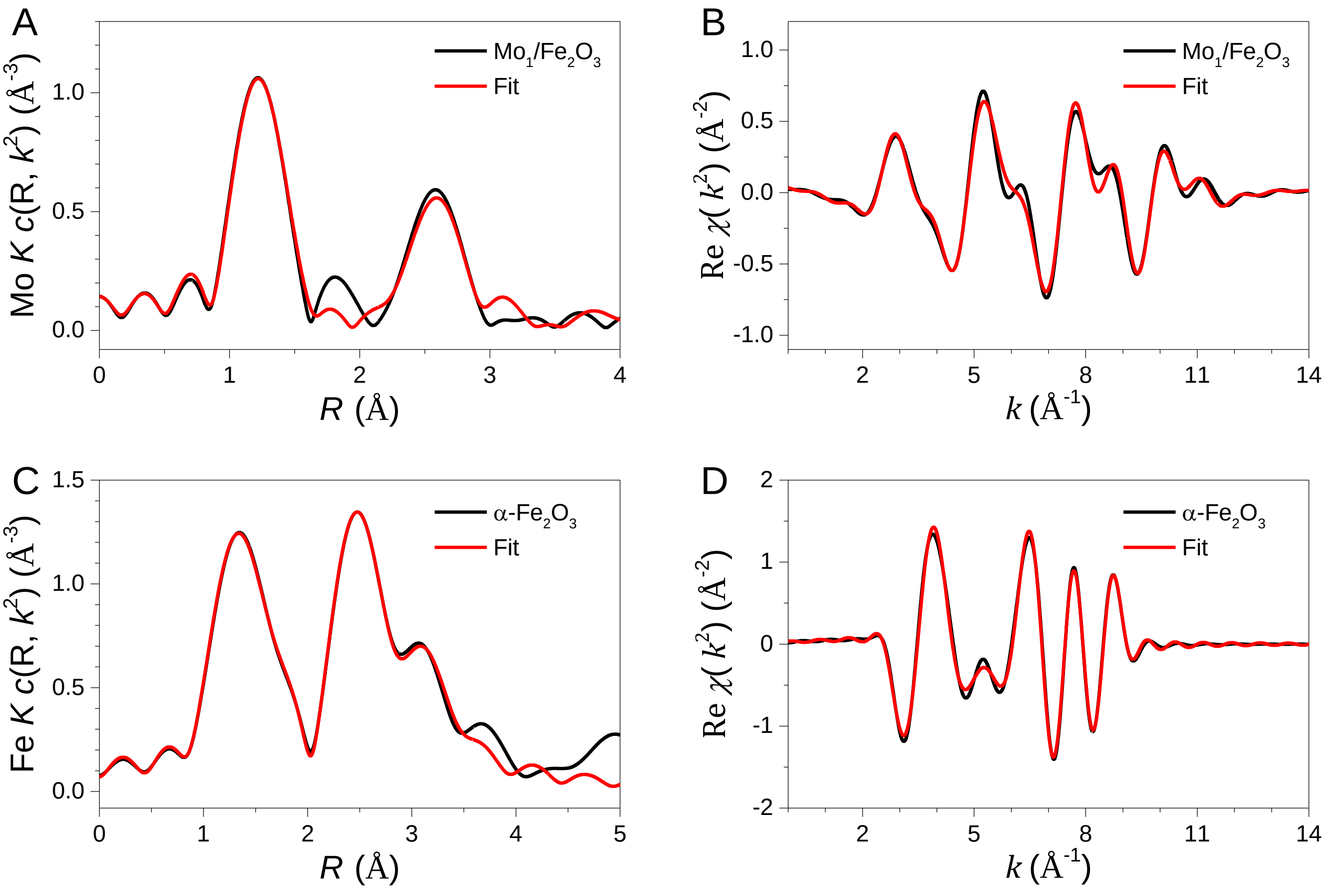

Supplementary Figure 5. (**A,B**) *R*-space (Δk = 2.3-11.4 Å^{-1}) and inverse FT spectra (Δr = 0.8-3.2 Å) at the Mo *K*-edge of Mo_1/Fe_2O_3. (**C,D**) *R*-space (Δk = 2.5-9.0 Å^{-1}) and inverse FT spectra (Δr = 0.4-4.1 Å) at the Fe *K*-edge of α-Fe_2O_3.

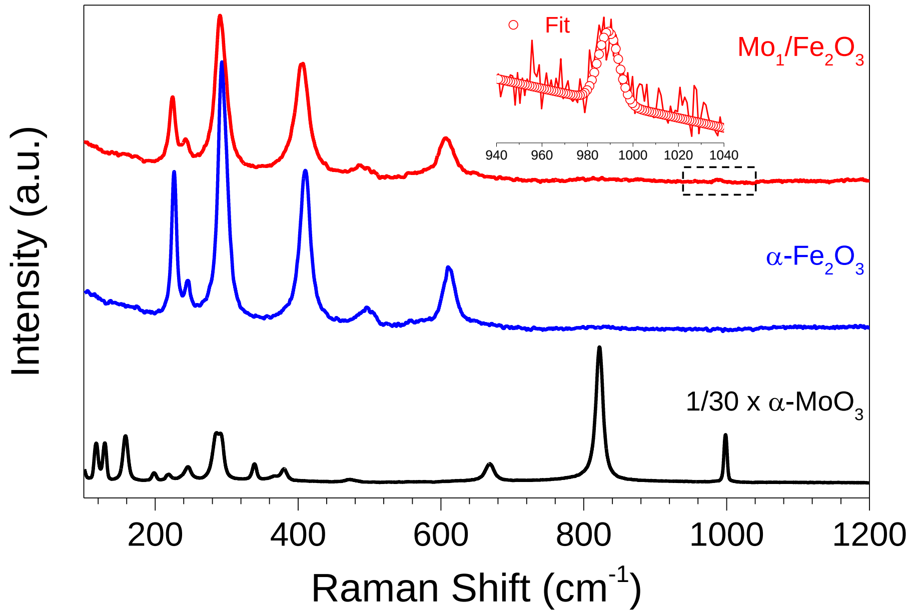

Supplementary Figure 6. Raman spectra of Mo_1/Fe_2O_3 (red line), α-Fe_2O_3 (blue line), and α-MoO_3 (black line). Inset: a double-bond-specific Raman band at ~989 cm^{-1} is ascribed to the Mo=O bond.

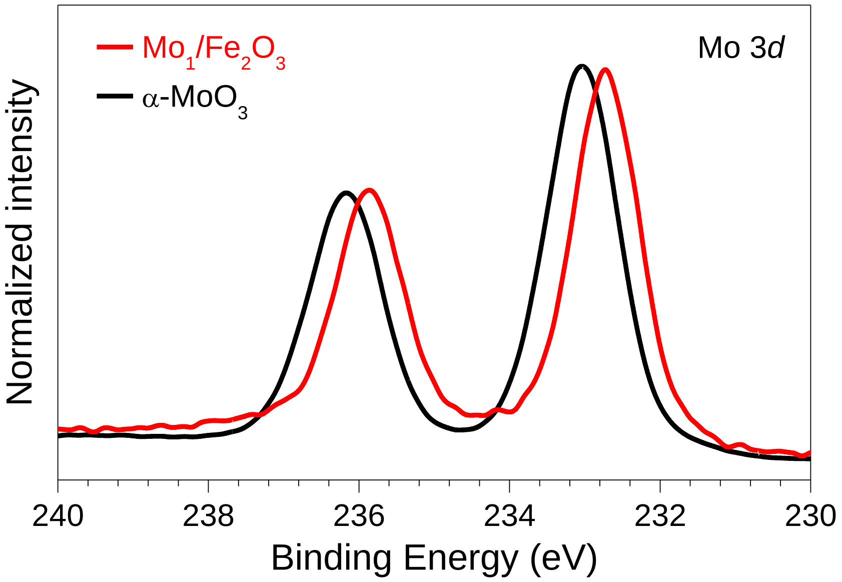

Supplementary Figure 7. X-ray photoelectron spectra of Mo 3*d* over Mo_1/Fe_2O_3 (red line) and α-MoO_3 (black line).

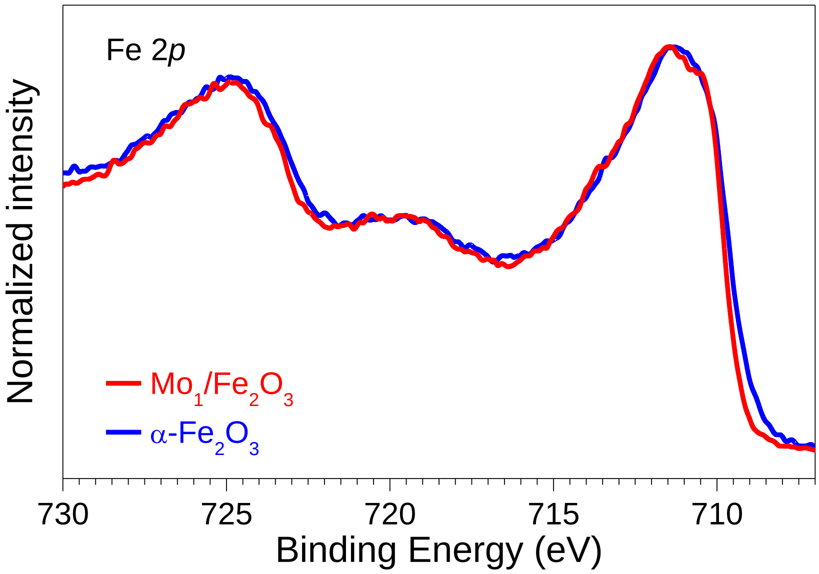

Supplementary Figure 8. X-ray photoelectron spectra of Fe 2*p* over Mo_1/Fe_2O_3 (red line) and α-Fe_2O_3 (blue line).

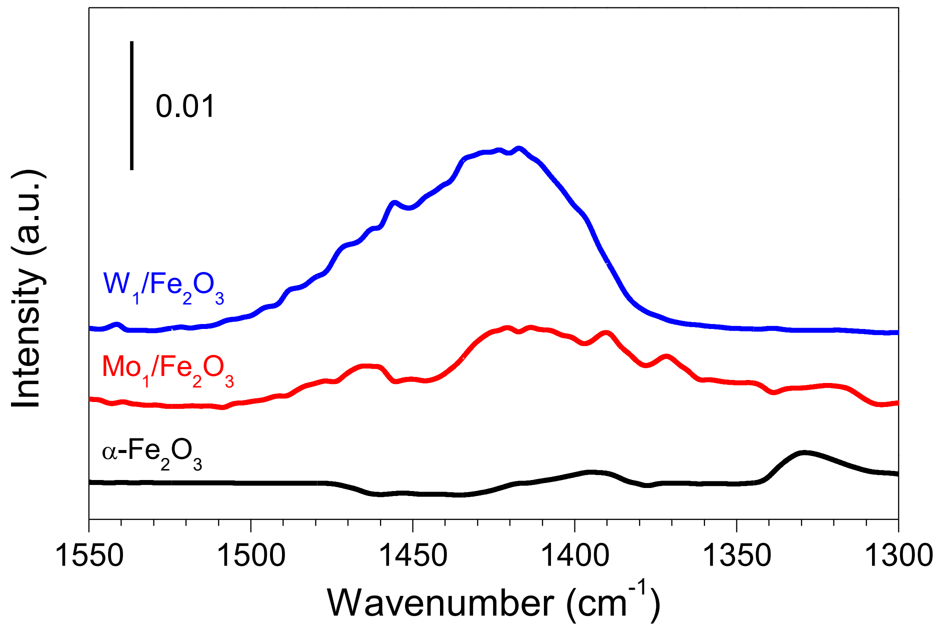

Supplementary Figure 9. DRIFT spectra of NH_3 adsorption on W_1/Fe_2O_3 (blue line), Mo_1/Fe_2O_3 (red line) and α-Fe_2O_3 (black line) at 50 °C.

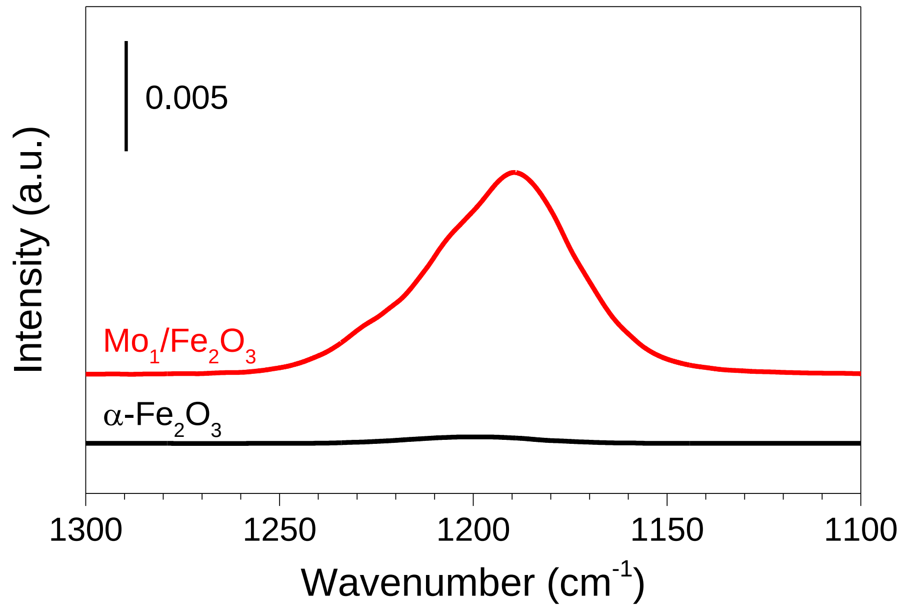

Supplementary Figure 10. DRIFT spectra of NH_3 adsorption on Mo_1/Fe_2O_3 (red line) and α-Fe_2O_3 (black line) at 250 ^oC.

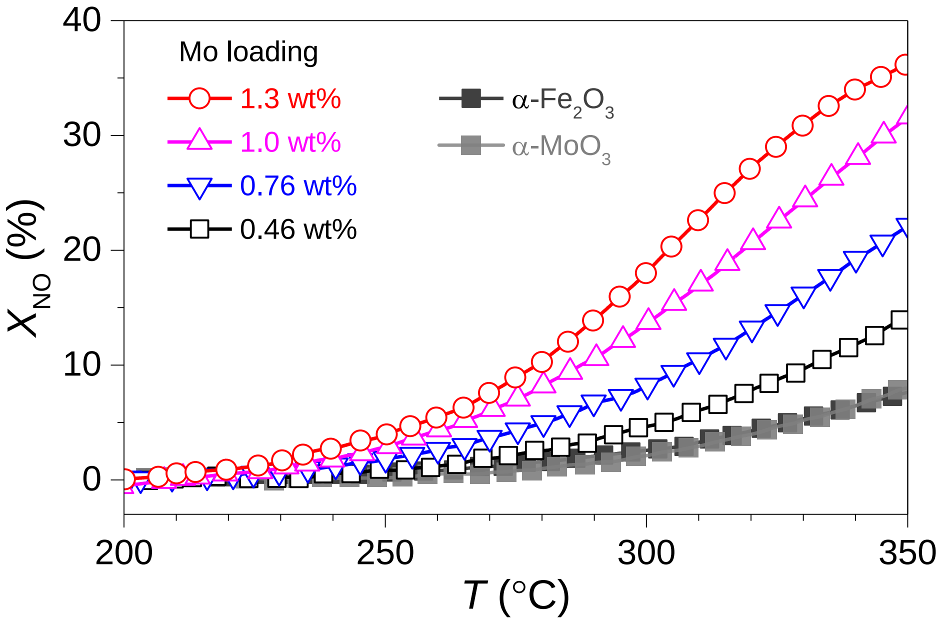

Supplementary Figure 11. X_{NO} as a function of temperature (T) over Mo_1/Fe_2O_3 with the different Mo loadings together with α-Fe_2O_3 and α-MoO_3. Reaction conditions: 500 ppm NO, 500 ppm NH_3, 3 vol% O_2, balance N_2, and GHSV 800,000 h^{-1}.

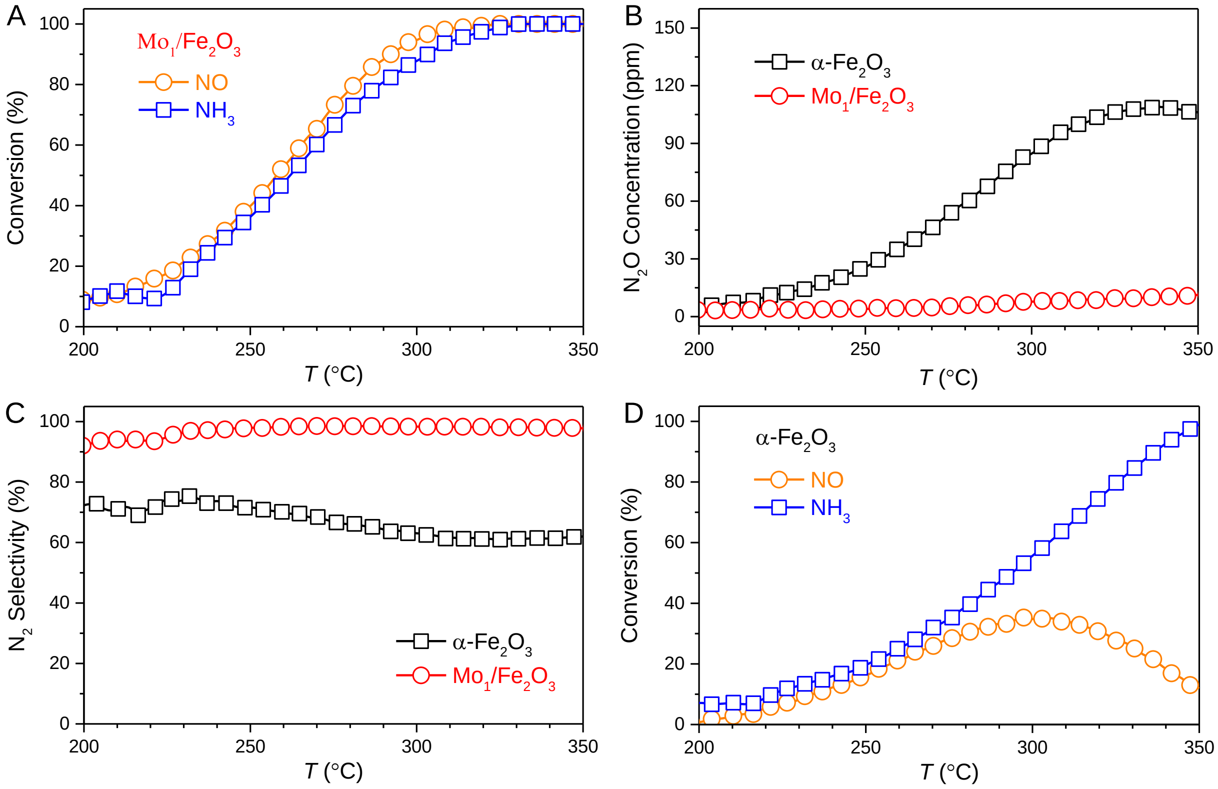

Supplementary Figure 12. SCR performance as a function of temperature (T): (**A**) NO (orange circle) and NH_3 (blue square) conversion over Mo_1/Fe_2O_3. (**B**) N_2O concentration over α-Fe_2O_3 (black square) and Mo_1/Fe_2O_3 (red circle). (**C**) N_2 selectivity over α-Fe_2O_3 (black square) and Mo_1/Fe_2O_3 (red circle). (**D**) NO (orange circle) and NH_3 (blue square) conversion over α-Fe_2O_3. Reaction conditions: 500 ppm NO, 500 ppm NH_3, 3 vol% O_2, balance N_2, and GHSV 66,000 h^{-1}.

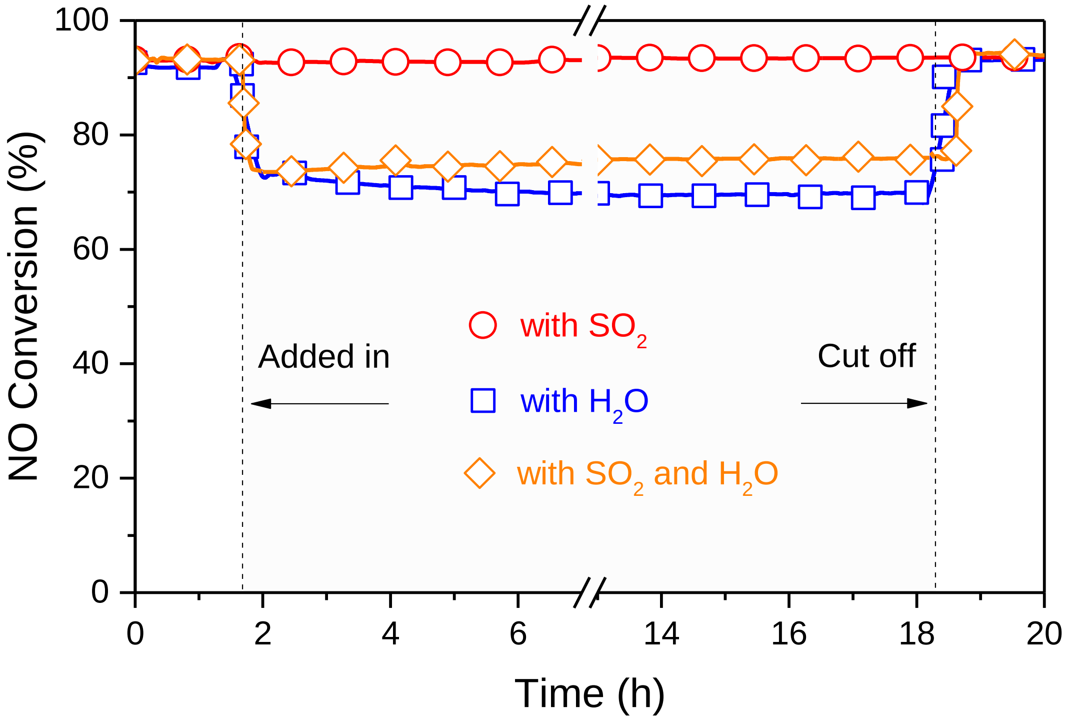

Supplementary Figure 13. Effect of H_2O and SO_2 on catalytic activity over Mo_1/Fe_2O_3 at 300 ^oC. Reaction conditions: 500 ppm NO, 500 ppm NH_3, 3 vol% O_2, 200 ppm SO_2 (when used), 5 vol% H_2O (when used), balance N_2, and GHSV 66,000 h^{-1}.

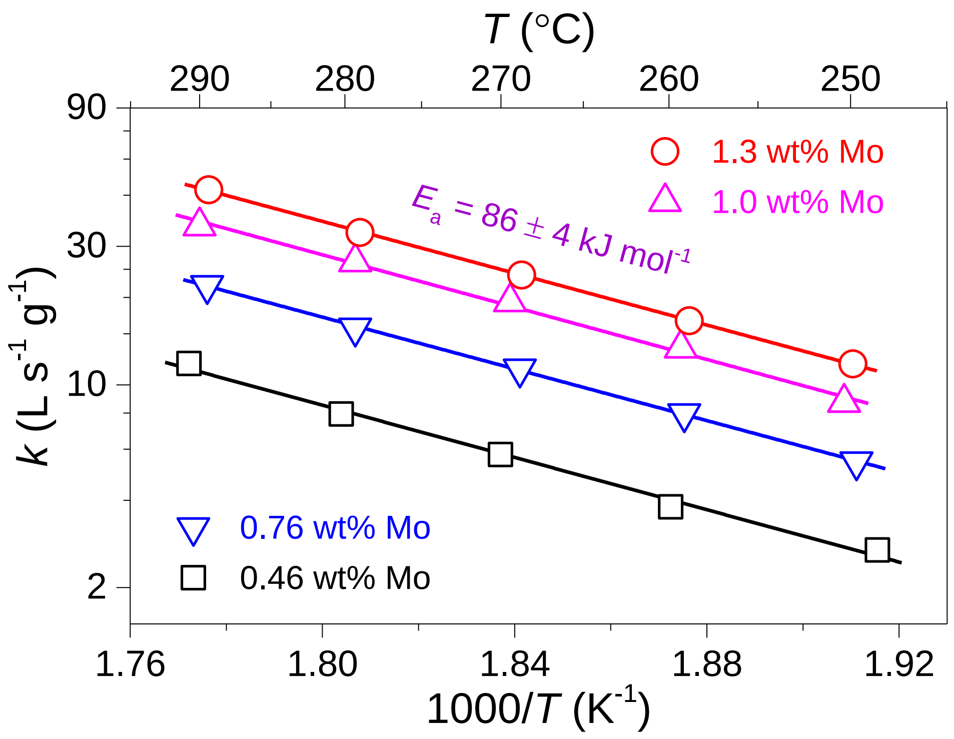

Supplementary Figure 14. Arrhenius plots of NO conversions in SCR over Mo_1/Fe_2O_3 with different Mo loadings. Reaction conditions: 500 ppm NO, 500 ppm NH_3, 3 vol% O_2, balance N_2, and GHSV 800,000 h^{-1}.

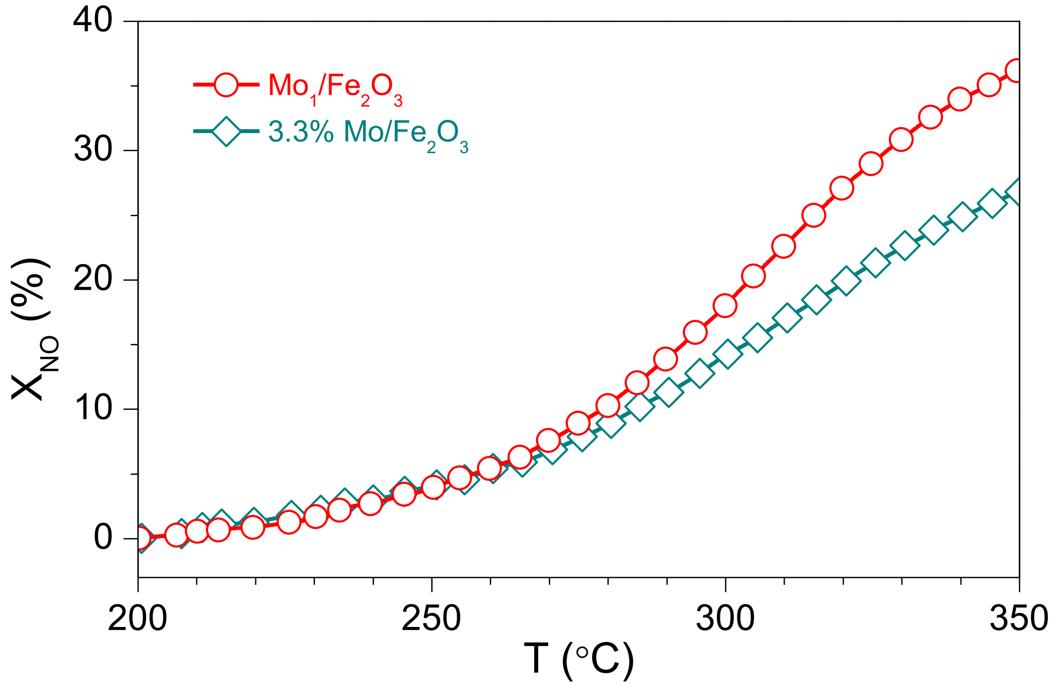

Supplementary Figure 15. X_{NO} as a function of temperature (T) over Mo_1/Fe_2O_3 (red circle) and 3.3% Mo/Fe_2O_3 (cyan square). Reaction conditions: 500 ppm NO, 500 ppm NH_3, 3 vol% O_2, balance N_2, and GHSV 800,000 h^{-1}.

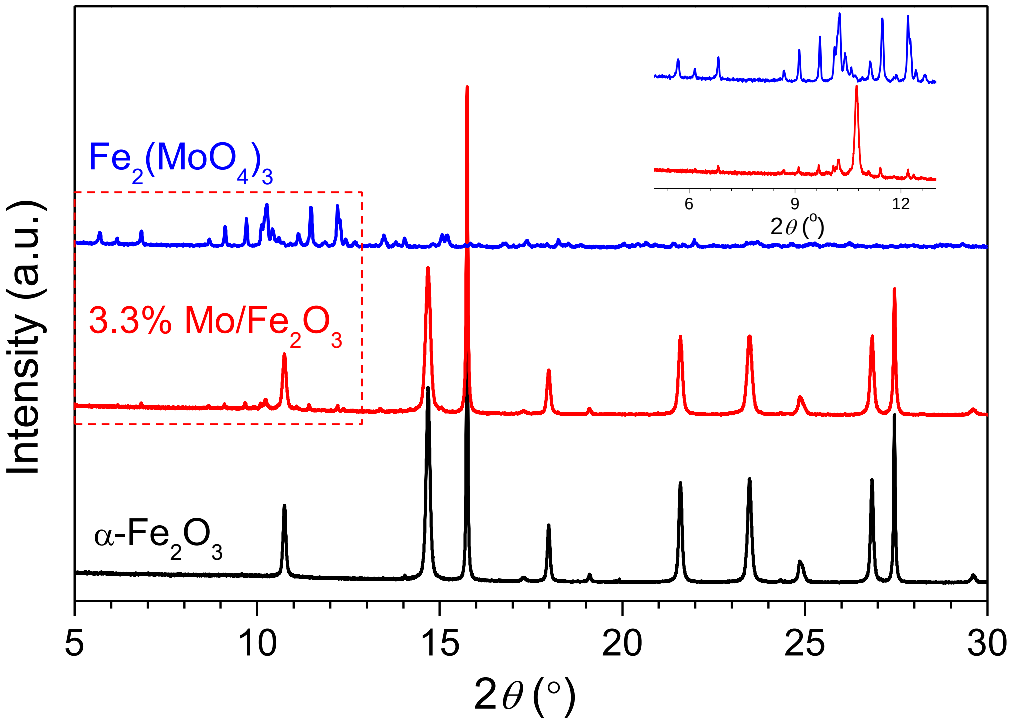

Supplementary Figure 16. SXRD patterns of $Fe_2(MoO_4)_3$ (blue line), 3.3% Mo/Fe_2O_3 (red line), and α-Fe_2O_3 (black line). Inset: the enlarged areas of the dashed rectangle showing diffraction due to the $Fe_2(MoO_4)_3$ phase on the surfaces of 3.3% Mo/Fe_2O_3

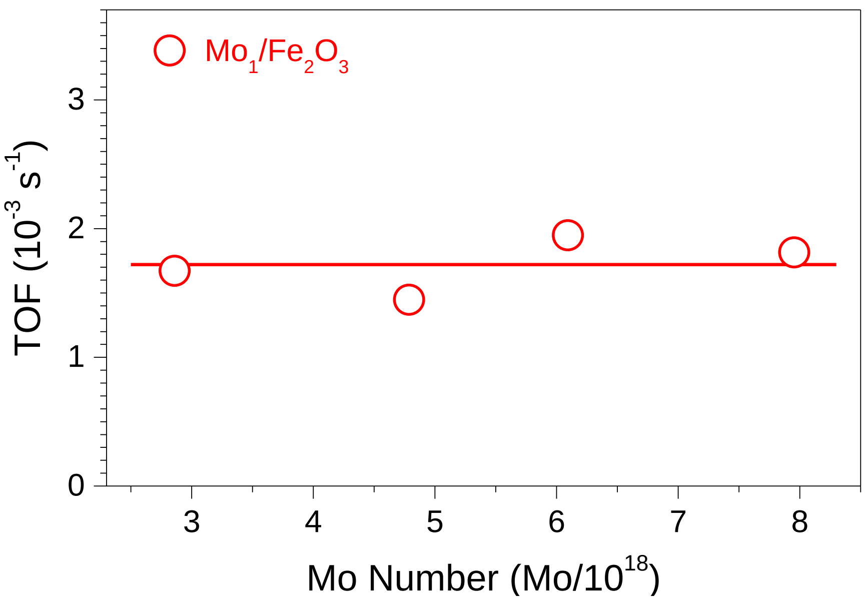

Supplementary Figure 17. TOFs in SCR at 270 ^{o}C over Mo_1/Fe_2O_3 with the different Mo loadings. Reaction conditions: 500 ppm NO, 500 ppm NH_3, 3 vol% O_2, balance N_2, and GHSV 800,000 h^{-1}. The red line is to guide the eye.

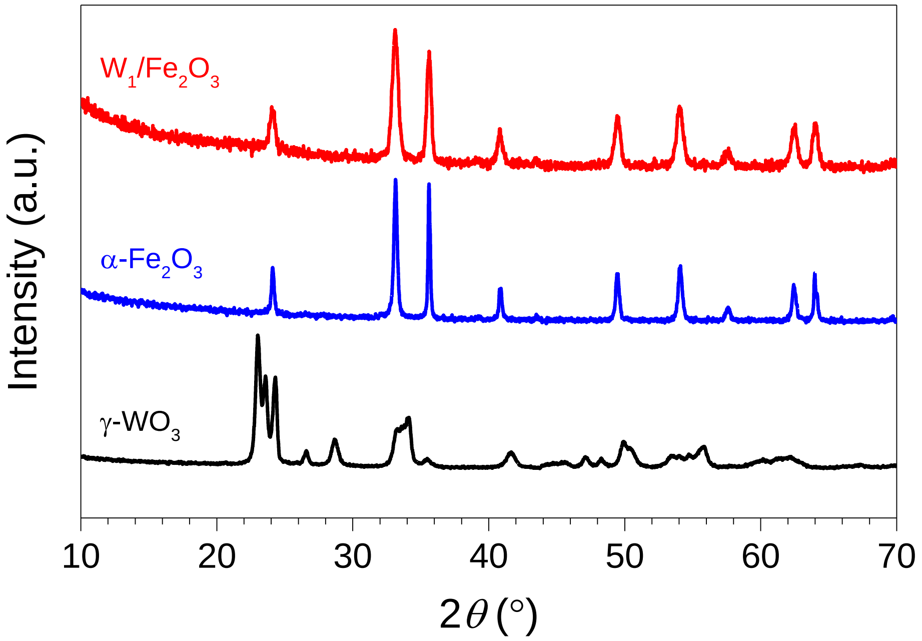

Supplementary Figure 18. XRD patterns of W_1/Fe_2O_3 (red line), α-Fe_2O_3 (blue line), and γ-WO_3 (black line).

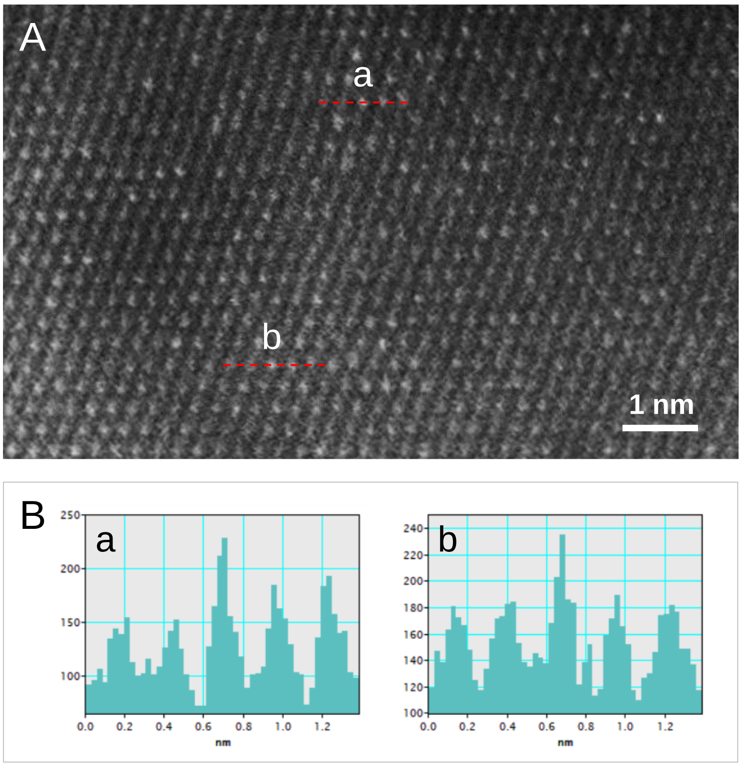

Supplementary Figure 19. (**A**) AC-STEM image of W_1/Fe_2O_3. (**B**) The image intensity line scans along the directions a and b shown in **A**.

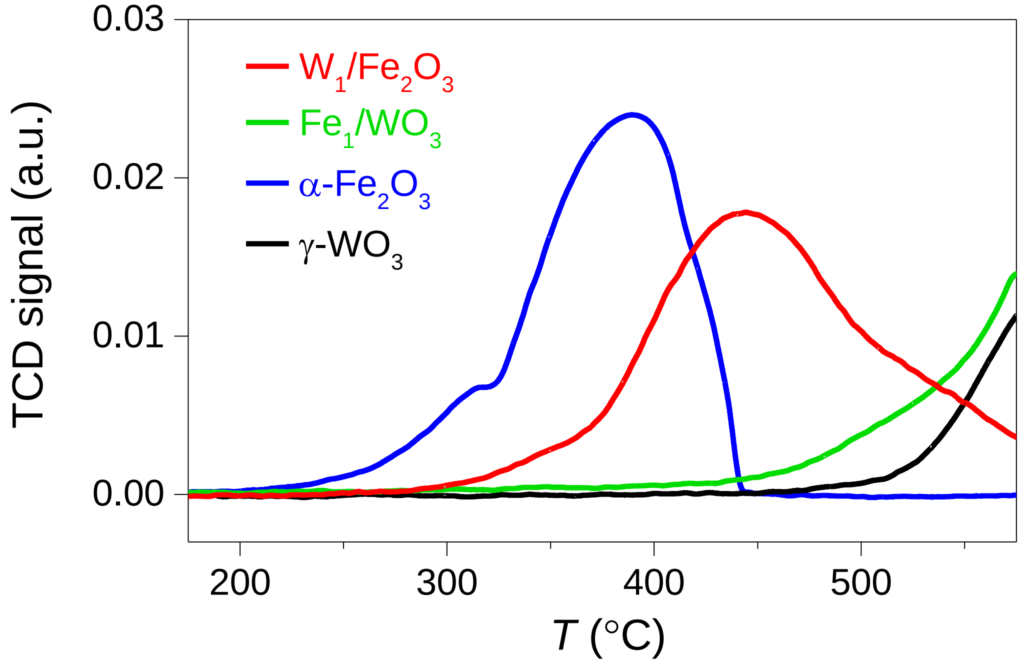

Supplementary Figure 20. H_2-TPR profiles of W_1/Fe_2O_3 (red line), Fe_1/WO_3 (green line), α-Fe_2O_3 (blue line) and γ-WO_3 (black line).

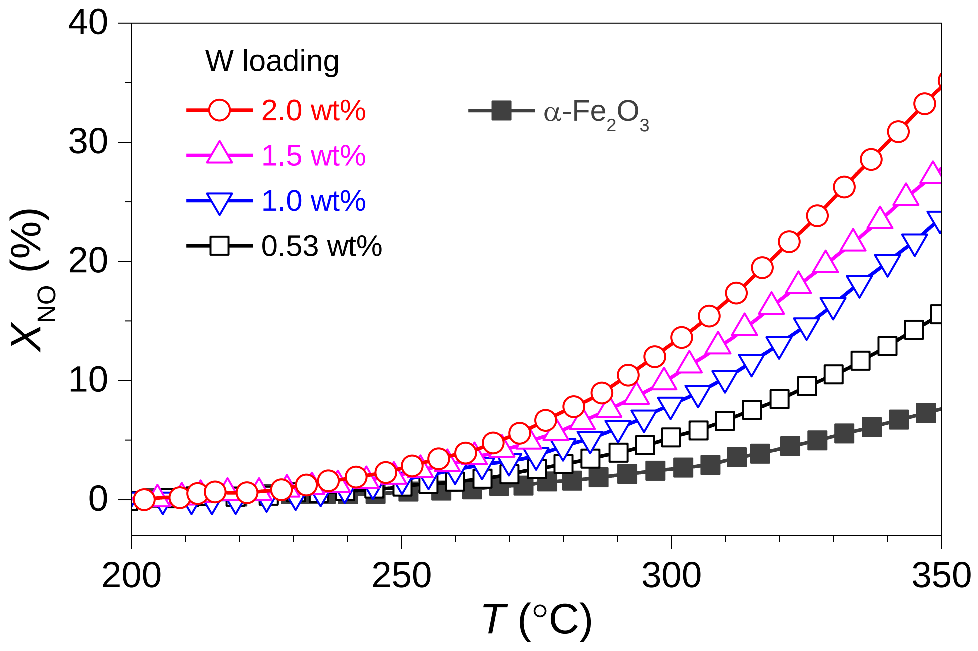

Supplementary Figure 21. X_{NO} as a function of temperature (T) over W_1/Fe_2O_3 with the different W loadings and α-Fe_2O_3. Reaction. conditions: 500 ppm NO, 500 ppm NH_3, 3 vol% O_2, balance N_2, and GHSV 800,000 h^{-1}.

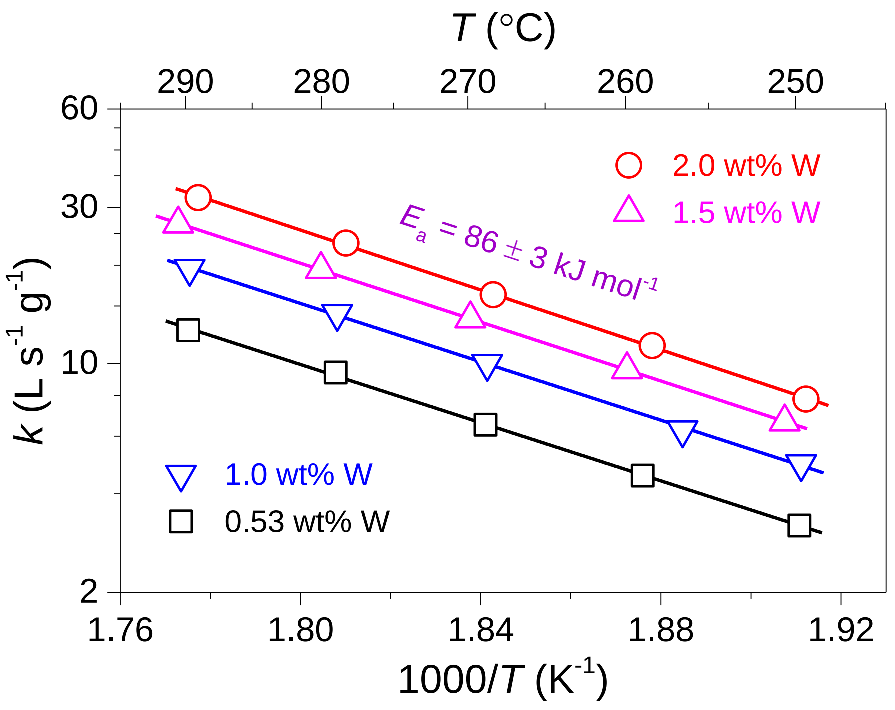

Supplementary Figure 22. Arrhenius plots of NO conversions in SCR over W_1/Fe_2O_3 with different W loadings. Reaction conditions: 500 ppm NO, 500 ppm NH_3, 3 vol% O_2, balance N_2, and GHSV 800,000 h^{-1}.

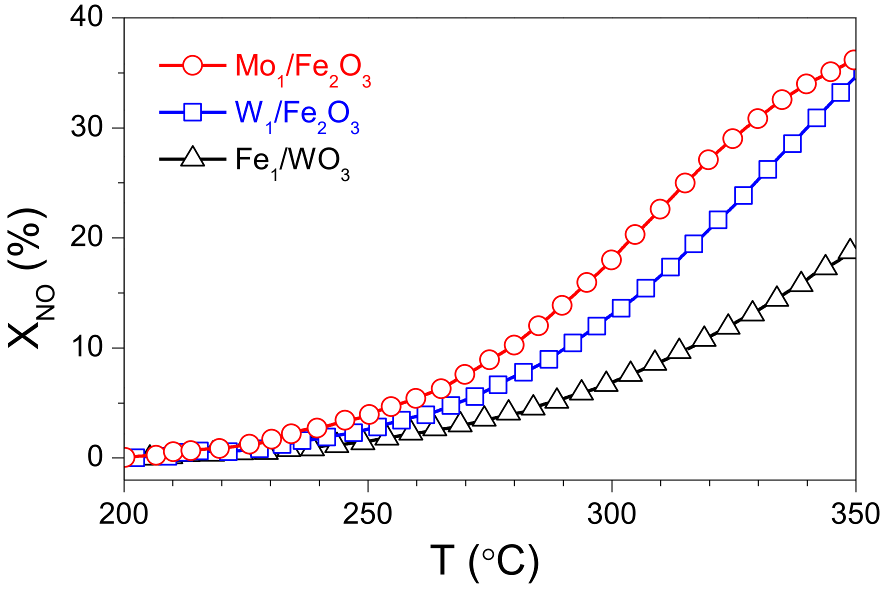

Supplementary Figure 23. X_{NO} as a function of temperature (T) over Mo_1/Fe_2O_3 (red circle), W_1/Fe_2O_3 (blue square), and Fe_1/WO_3 (black triangle). Reaction conditions: 500 ppm NO, 500 ppm NH_3, 3 vol% O_2, balance N_2, and GHSV 800,000 h^{-1}.

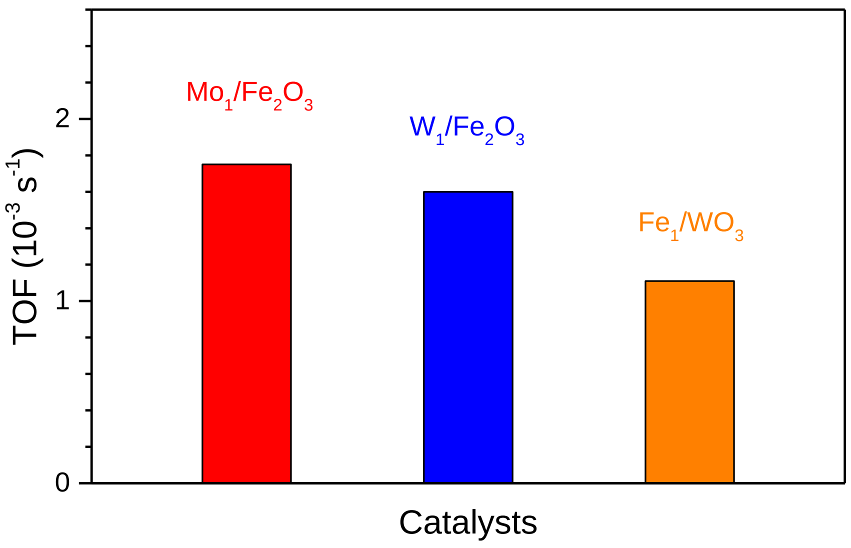

Supplementary Figure 24. TOFs over Mo_1/Fe_2O_3 (red), W_1/Fe_2O_3 (blue), and Fe_1/WO_3 (orange) at 270 °C. Reaction conditions: 500 ppm NO, 500 ppm NH_3, 3 vol% O_2, balance N_2, and GHSV 800,000 h^{-1}.

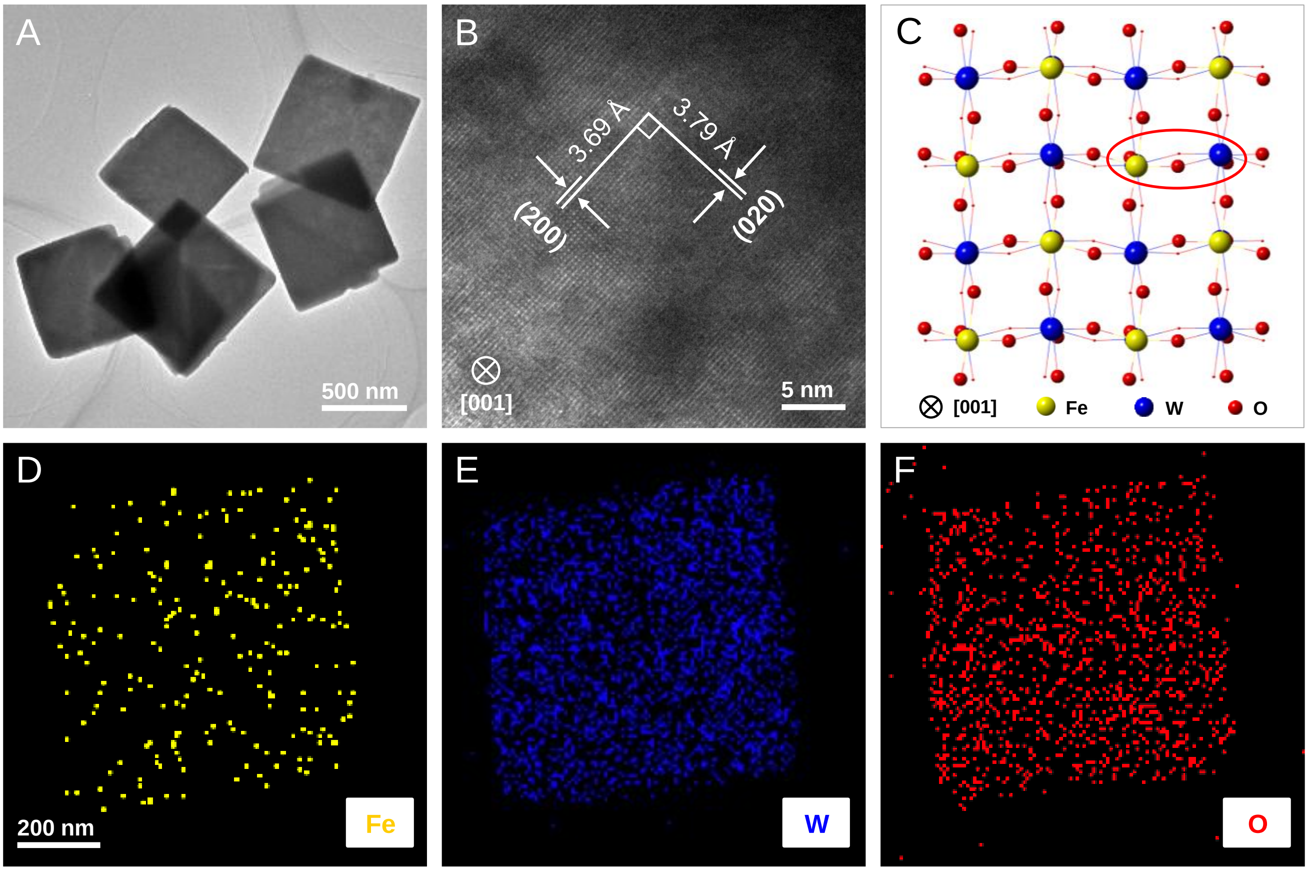

Supplementary Figure 25. (**A**) TEM and (**B**) HRTEM images of Fe_1/WO_3. (**C**) Structural model of Fe_1/WO_3. The yellow, blue and red balls represent Fe atoms, W atoms, and O atoms, respectively. The typical Fe_1-W_1 dinuclear site is marked by red ellipse. (**D-F**) EDX mappings of Fe_1/WO_3.

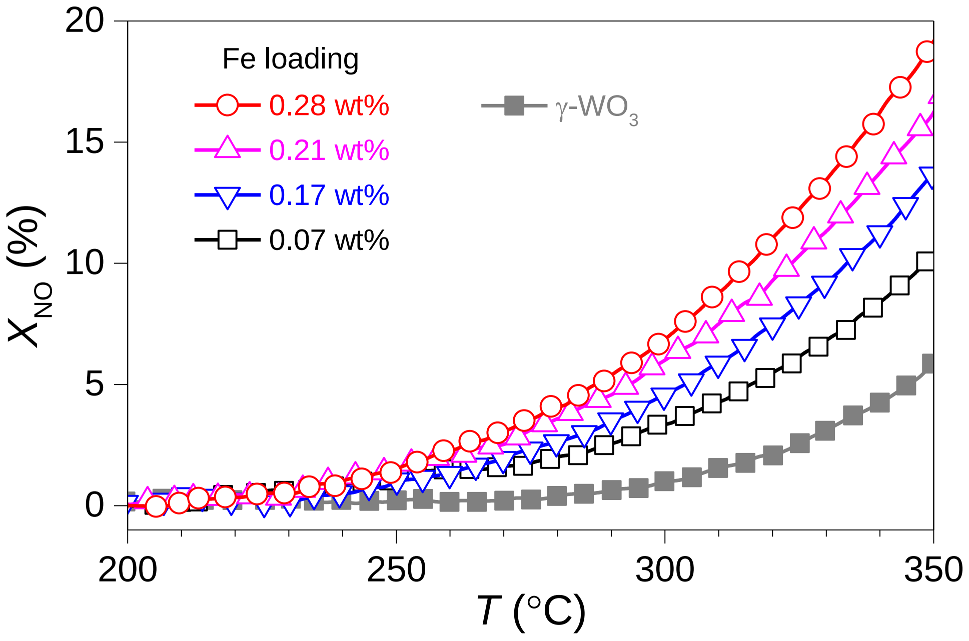

Supplementary Figure 26. X_{NO} as a function of temperature (T) over Fe_1/WO_3 with the different Fe loadings and γ-WO_3. Reaction conditions: 500 ppm NO, 500 ppm NH_3, 3 vol% O_2, balance N_2, and GHSV 800,000 h^{-1}.

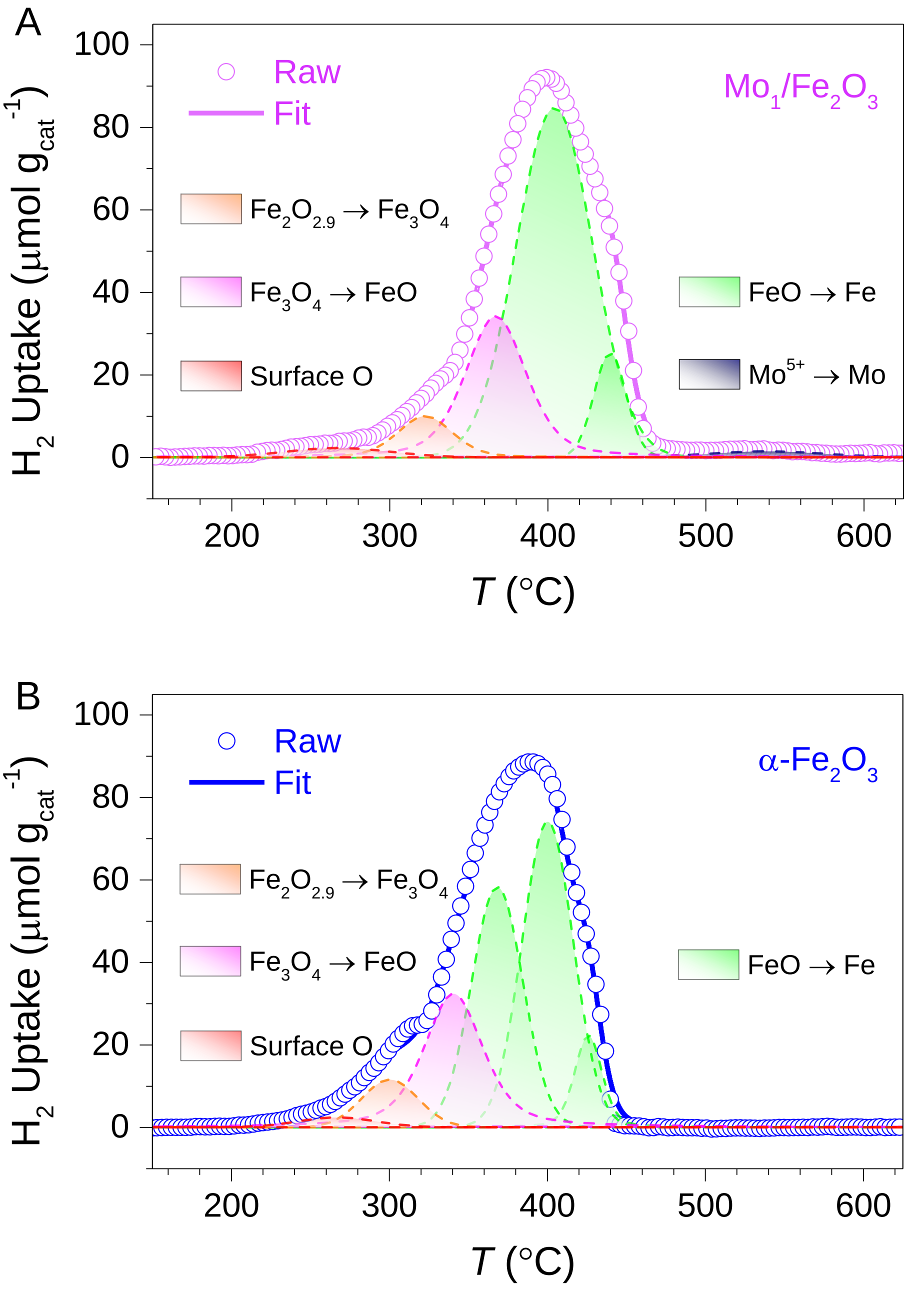

Supplementary Figure 27. The deconvoluted H_2-TPR profiles of (**A**) Mo_1/Fe_2O_3 and (**B**) α-Fe_2O_3.

Supplementary References

1. Hartel, P., Rose, H. & Dinges, C. Conditions and reasons for incoherent imaging in STEM. *Ultramicroscopy* **63**, 93-114 (1996).

2. Ortalan, V., Uzun, A., Gates, B. C. & Browning, N. D. Towards full-structure determination of bimetallic nanoparticles with an aberration-corrected electron microscope. *Nat. Nanotechnol.* **5**, 843-847 (2010).
